# Click Reactions in Medicinal Chemistry

**DOI:** 10.3390/ph16101361

**Published:** 2023-09-27

**Authors:** Damien Bosc

**Affiliations:** Institute Pasteur Lille, U1177-Drugs and Molecules for Living Systems, Univsity Lille, Inserm, F-59000 Lille, France; damien.bosc@univ-lille.fr

Two decades after K. B. Sharpless introduced the term “click chemistry”, its profound influence on the field remains palpable [1]. Click reactions, and especially the CuAAC, with their specific attributes, present an ideal toolkit for the multifaceted demands of drug discovery. In this pursuit, the ability to perform click reactions within biological systems has become particularly attractive to researchers aiming to interface chemistry with biological macromolecules. This partnership expedites the creation of diverse compound libraries and chemical probes, addressing the need for efficiency and innovation in drug development. Particularly remarkable is click chemistry’s application within biological contexts, opening doors to chemical exploration within intricate biological macromolecules.

At the forefront of click chemistry’s impact on medicinal chemistry is the CuAAC. Its advantages—high efficiency, modularity, and facile access to 1,4-disubstituted 1,2,3-triazoles—make it the quintessential choice for medicinal chemists [2]. But CuAAC is just the tip of the iceberg. Click reactions such as cycloadditions, nucleophilic substitutions, and sulfur-fluoride exchange [3] have augmented the medicinal chemist’s arsenal, expanding the horizon of possibilities.

This special issue of Pharmaceuticals delves into the expansive landscape of click chemistry in medicinal applications, showcasing four diverse articles and one review that shed light on its potential.

Guo et al. delved into the design and synthesis of novel pomalidomide derivatives containing urea moieties. These compounds exhibited significant anti-tumor potential in vitro, particularly against breast cancer cell lines. Notably, one compound displayed remarkable inhibitive activity and induced cell death through ROS elevation and DNA damage. This study paves the way for further investigations into these derivatives as potential anti-tumor agents [4].

The second article from Hou et al. centers on the exploration of IDO1 inhibitors, which are crucial in the realm of cancer immunotherapy. Through innovative design and synthesis of urea and 1,2,3-triazole-containing compounds, the authors unveiled a promising inhibitor. Molecular docking provided insights into the compound’s interaction with IDO1, fueling the development of IDO1 inhibitors [5].

Oekchuae et al. present and discuss the development of anti-HCC drug structures. Through the integration of 1,2,3-triazole-cored structures with aryl urea moieties, the authors synthesized compounds with improved selectivity indexes compared to existing drugs. These promising candidates demonstrated interesting anti-HepG2 activity and appropriate drug-likeness, positioning them as good starting points to develop potential therapeutic agents [6].

García-Vázquez et al. reviewed the current state-of-the-art of the burgeoning field of theranostics by focusing on tetrazine ligation in nuclear medicine. Pretargeting strategies using tetrazines offer the potential to revolutionize radiopharmaceuticals by enhancing target-to-background ratios, thus optimizing therapeutic and diagnostic approaches. This review underscores the significance of tetrazine-based strategies in shaping the future of precision medicine [7]. In this context, Lopes van den Broek et al. present their research on this technique with profound implications for neurodegenerative diseases. By leveraging bioorthogonal chemistry, the authors explore the feasibility of imaging currently ‘undruggable’ targets. Their work provides crucial insights into achieving sufficient imaging contrast in pretargeted autoradiography, offering promise for monitoring therapies for challenging neurodegenerative conditions [8].

The compilation of these articles underscores the profound impact of click chemistry in medicinal applications. From novel anti-tumor agents to innovative imaging techniques, the versatility of click reactions is evident. These studies not only expand our understanding of molecular interactions and disease mechanisms but also open new avenues for therapeutic development. As we navigate the intricate landscape of medicinal chemistry, this Special Issue has shown that the integration of click chemistry continues to foster innovation and redefine possibilities. The articles presented in this Special Issue exemplify the collaborative efforts of researchers pushing the boundaries of drug discovery.
